# Round the Hospitals

**Published:** 1918-07-13

**Authors:** 


### ROUND THE HOSPITALS.

ON July 6, 1918, the words " happiness and contentment " aptly described the state of mind and joyous feelings of their Majesties' subjects resident in the Metropolis of the Empire, and throughout the Empire, and of our fighting men of all ranks at the seats of war. Elsewhere we have shown how closely this joyous feeling was shared with their subjects by the King and Queen at their Silver Wedding. This knowledge recalls to our memory an incident recorded in connection with their Majesties' wedding-day. Queen Mary's mother, the late Princess Mary of Cambridge, whose happy temperament won for her the love of all who had the privilege of knowing her, was full of joy on her daughter's marriage. After the ceremony, when she found herself at Buckingham Palace

**ROUND THE HOSPITALS—(*continued*).**

with our beloved Queen, the late Queen Victoria, it is recorded that she danced about with joy and brought happiness and high spirits to everyone present. After the twenty-five years which have elapsed, the celebration of the Royal Silver Wedding on Saturday by their Majesties and their subjects fufilled the joyous presentiment which moved Queen Mary's mother to give expression to it in the way we have just described. It is well for the people of all classes to treasure this incident and presentiment and its fulfilment, for it typifies with attractive spontaneity the soundness of the English feeling that the world provides nothing so perfect, uplifting, and of such life-long importance as the ideal home, which is one of the joyous fruits of their Majesties' marriage and its consummation.

Amongst the deputations with addresses of congratulation on their Silver Wedding which the King and Queen, accompanied by Princess Mary, who was in V.A.D. uniform, and Prince Albert, wearing the uniform of the R.A.F., received on Monday, the 8th inst., at Buckingham Palace, that presented by the London County Council has a special and peculiar interest for our readers. It called forth from King George the statement that "I inherited from my dear father a profound interest in the splendid work of the London hospitals, and I rejoice that in spite of the adverse effect of the war upon their financial resources, these national institutions have been able to continue and even to extend their mission of charity." King George has nobly fulfilled this inheritance by setting a splendid example of personal service in the days of health in the cause of the sick, which he has continuously rendered year by year with deliberate purpose as one result of the profound interest which he inherited from our late beloved King, King Edward VII. The voluntary hospitals are for the people of England a precious inheritance in fact. To them all classes owe a hospital system, the first aim and object of which is always the welfare and benefit of the patient, worthy of our freedom as a nation and Empire, and our united upholding of the principles for which we are fighting in the present war.

Further particulars which have come to light on the sinking of the hospital ship *Llandovery Castle* reveal the poignant fact that one of the murdered nurses, Sister Margaret Marjorie (Pearl) Fraser, had on many occasions tended the German wounded and rendered them kindly services outside the ordinary routine, when on duty in casualty clearing-stations during the past three years. "Many times had she been the first to give a drink of water to these parched enemy casualties. Many a time had she written down the dying statements of enemy officers and men, transmitting same to their relatives through the Red Cross organisation." Sergeant Knight, R.A.M.C., who had charge of the lifeboat in which the fourteen nurses were placed, gives a thrilling account of the catastrophe which overwhelmed them. They were in the boat for about eight minutes before it was sucked under. All were perfectly calm and collected, perfectly *conscious*. As the boat was drawn in towards the stern of the ship, the matron, Sister M. M. Fraser, asked if there were any hope for them. Sergeant Knight replied that there was none, and almost as he spoke they were in the whirlpool of the sinking ship, and all were thrown over the side of the boat. All wore life-belts, but it must be doubtful if they ever rose again to the surface. Two were in their night attire, the rest in uniform.

The names of the sisters who lost their lives in this manner were Matron Margaret M. Fraser, Sisters Christine Campbell, Carola J. Douglas, Alaxina Dussault, Minnie Follette, Margaret J. Fortescue, Minnie K. Gallaher, Jessie M. McDiarmid, Mary A. McKenzie, Rena McLean, MacBelle Sampson, Gladys I. Sare, Anna I. Stamers, Jean Templeman. Most of them volunteered for service in 1914 when war broke out, and had been employed in casualty clearing-stations until they were transferred to transport duty by way of affording them a rest. It appears to have been only by accident that there were any survivors of the *Llandovery Castle*. The submarine's commander unquestionably aimed at destroying everyone of the party in order that no record of his deeds might be made public. We rejoice to note that the Pope has been moved by the tale to issue a horrified remonstrance.

The Western Infirmary, Glasgow, has made a new departure by starting a school of massage where students receive the necessary training in preparation for the examination held by the Incorporated Society for the certificate of membership. As there is serious demand for the services of trained masseuses for the treatment of disabled sailors and soldiers, and as up till now there has been no school attached directly to any hospital in Scotland, the Western Infirmary authorities have done good service by making the provision just mentioned. The first course began early in January, and the first written and practical examinations for all the Scotch students were held in June at the Western Infirmary. It is a great advantage for students on the other side of the Border to be able to have their examination in Glasgow instead of going to one of the English centres. Thus the development and extension of the College of Nursing, the facilities for probationers and trained nurses, and the best interests of both are extending rapidly and satisfactorily in Scotland under new and up-to-date conditions.

As this week's issue reaches the hands of our readers London, with many other parts of the country, will be making its annual effort on behalf of the Croix Rouge Française. France's Day will be enlivened by the presence of the premier Zouave Band of the French Army, with a guard of honour, numbering in all seventy men, from the pro-

**ROUND THE HOSPITALS—(*continued*).**

vince of Alsace-Lorraine. With an admirable pre-war organisation the Croix Rouge Française has always carried on its devoted work for the wounded under the greatest difficulties by reason of the colossal demands on its too scanty resources. Without increased British support its activities must, we fear, be curtailed rather than extended. A record collection would relieve much anxiety for the wounded of our great Ally.

There is no more promising local Centre of the College of Nursing than that at Bristol. It was inaugurated in March last at a meeting called by Miss Baillie, matron of the Royal Infirmary, and the committee then formed included representatives from the Bristol General Hospital, the 2nd Southern Hospital, Bristol Royal Infirmary, Southmead, Redmaids, Queen Victoria Jubilee Institute, Bristol Nurses' Institution, Ham Green Fever Hospital, Bath Royal United Hospital, Gloucester Royal Infirmary and Eye Institute, Cheltenham General Hospital, Taunton and Somerset Hospital, Children's Hospital, St. Michael's Hill, Bristol. The list gives a vivid picture of Bristol's situation as a meeting ground and of the influential support enlisted from the outset. The officers appointed were Miss Baillie, chairman and local representative; Miss McHardy, hon. secretary; Miss Densham, hon. treasurer. A finance committee is being formed. Dr. Lily Baker has given the first lecture to the Centre—on Infant Welfare Work. All promises well for a brilliant session after the holidays.

The preliminary business of organisation having been accomplished, Bristol was ripe for a public meeting to make known to a wider circle the aims of the College of Nursing. Accordingly, on July 5 the Hon. Sir Arthur Stanley came down to address an influential gathering at the Museum Lecture Theatre, with the Lord Mayor in the chair. He developed the paramount importance of protecting the public from ill-qualified nurses, and of protecting nurses from the risks they incurred in the course of their work, both of which purposes the College aimed at fulfilling. He touched on the difficulties experienced with regard to nurses trained at hospitals too small to be recognised as training-schools, and expressed the hope that the smaller hospitals might, through the agency of the College, be divided into groups in order that the training might be rendered complete. He informed the meeting that the College had now ten local Centres; the management was centred in London, but each Centre would enjoy perfect freedom and self-government within its own limits. They must all be self-supporting, and would doubtless contribute in time to the Central Endowment Fund. Major Patrick Watson Williams and Major Lansdown followed with excellent speeches.

Miss Baillie, whose words were received with enthusiasm, concluded with a succinct account of the idea which underlies the College, its chief aims, its post-graduate courses and scholarships, its future bright with promise, and demonstrated that all was included in the one great purpose for which the art of nursing had been developed. "It is all," she said, "that we may become more helpful to the sick." Many new members gave in their names after the meeting, and all members of the College within the sphere of this Centre should at once communicate with Miss J. M. McHardy, 4 Chesterfield Place, Clifton, Bristol. Nurses who are not already members of the College should write to the Secretary of the College of Nursing, 6 Vere Street, London, W. 1, for papers.

We are asked to state that Chesterfield and district are included in the new local Centre of the College of Nursing started at Derby. The Chesterfield and North Derbyshire Hospital, with its 120 beds, forms one of the important nurse-training centres of the county.

One of the most successful efforts yet made on behalf of the Nation's Fund for Nurses took place on July 9 in the beautiful gardens of St. James's Palace, graciously lent for that purpose by the King. Nothing could have been devised more picturesque and graceful than this sumptuous garden party, where gay little plays, interspersed with dancing, singing, and mumming, made up a constantly moving panorama. A crowded audience, among whom was Princess Patricia, assembled to enjoy the fête. Stalls laden with fruit and flowers and tempting trifles lent an added attraction, and brought much grist to the mill. The evidence afforded by the occasion of the King's approval and his full sympathy with the objects of the Fund was perhaps even more welcome to its promoters than the rich harvest gathered in towards the Nation's Tribute.

The Westminster Hospital, in its recently issued annual report, shows evidence of the effect of war upon the training-schools. There were but 320 applications during the past year from candidates desirous of being trained as nurses, a diminution of 148 on the previous year, and about half the old average. A striking shrinkage in the number of nurses on the private staff also reveals war-time conditions. In 1914 there were between seventy and eighty on the private staff, and nurses were promoted to its ranks from the hospital staff at the rate of about twelve a year. In 1917 it numbered thirty-five. A very large number of these nurses are of course now doing naval and military service, and the hospital has never had more reason to be proud of its private staff. The Nursing Board report that the Lady Superintendent, Miss Edith Smith, has been honoured by the receipt of the Royal Red Cross, and this decoration has also been conferred on Nurse Reynolds Knight. The work of the Westminster Training-School has been carried out of late years under peculiar difficulties, since the sands of the historic old building are fast running out, and accommodation has long

**ROUND THE HOSPITALS—(*continued*).**

been outgrown. Nevertheless, the work of training nurses, however it may have been complicated in a building constructed in days when modern methods were unknown, displays no deterioration. The outside examiner, Mr. Russell Howard, M.S., surgeon to the London Hospital, passed seventeen candidates of the nineteen who took their final last year, and of these two were "excellent" and nine "very good." It is certain that women trained to overcome perpetual problems presented to them by want of space and modern apparatus become exceptionally well qualified to overcome such problems as may assail them in private work, in municipal nursing, and under war conditions.

An important agreement has been entered into by the authorities of the City of London Hospital for Diseases of the Chest, Victoria Park, with the Great Northern Central Hospital. Two years' training at the Victoria Park Hospital is now accepted by the Great Northern Central Hospital as the equivalent of one year's general training. Nurses will therefore, after their two years' course at the special hospital, be given the status of second-year probationers when they are passed on, and be spared the humiliation (and we may add the waste of effort) involved in beginning all over again as novices after passing stiff examinations preceded by a carefully-thought-out curriculum of study. The scale of salaries has at the same time been substantially increased at the City of London Chest Hospital. Probationers now receive £20 in their first year and £22 in their second, with the usual allowances. Both the rise in salaries and the care taken to facilitate after-training are likely to attract good probationers and raise still higher the standard of training in the institution.

A severe rebuke has been administered by Judge Hand in the Federal District Court at New York to Dr. Emma Culbertson, senior surgeon of the New England Hospital for Women and Children at Boston, Mass., for statements made at Vassar College, seriously reflecting on the morals of the American Red Cross nurses in France. We abstain from repeating the slanders, which were of a particularly odious character. Happily, like the equally odious slanders industriously circulated against one of our own Women's Corps, they were of a nature which it was easy to disprove. The grand jury were asked to go carefully into the truth of the statements made, since the slanders, if true, constituted a case of gross neglect on the part of the army authorities, and, if false, they exposed the person who disseminated them to a conviction under the Espionage Act. Dr. Culbertson admitted that she had absolutely no knowledge or information on which to base her statement, and that it was entirely without foundation. She was absolved from vicious intent, but the judge declared her statements to have been "most reprehensible and disgraceful." So fatally tempting is the process of handing on a slander that we deem it to be the duty of any who hear evil talk directed against war-workers in the present juncture to raise a protest sufficiently stern to frighten the delinquents for evermore into respect for the Ninth Commandment.

The agitation against the fourth year of training and the overworking of nurses in hospitals continues, writes our Australian correspondent, and is not likely to cease. At the end of April the State Cabinet decided that before taking action an independent investigation of the complaints should be made. Mr. Meek, Chief Clerk of the Treasury, has been appointed to conduct the inquiry and submit a report to the Cabinet. The nurses' complaints are not by any means confined to the extra year and the long hours, but include badness and insufficiency of food, lack of time for study and attending lectures, and above all the preponderance of the matron's influence and opinion, against which there is no appeal. It is alleged that a word from the matron may wreck the nurse's whole career, and this "absolutism" is deemed to be undemocratic and un-Australian.

New Zealanders in England have started a Voluntary Aid Detachment for which New Zealand girls living in England who do not belong to a Detachment in their own country are eligible. The Commandant is Miss Helen Mackenzie, daughter of the High Commissioner, and the headquarters are at 11 Southampton Row, W.C. 2. All the Dominions, as well as the British West Indies, have now provided themselves with a voluntary corps of workers in aid, and America has done the same. The demand for the services of these voluntary aids continues unabated, and can only be met by making full use of what may be called the 1918 Class, viz. the girls leaving school this year. They are almost as necessary to the nation at this moment as their brothers.

The governors of the North Devon Infirmary have elected two members of the Ladies' Association to act on their committee. It is a welcome testimony to the benefits derived from this association, which has been at work since 1904, and held its fourteenth annual meeting on June 28. There are now seventy-nine members, who contribute a yearly subscription in addition to two articles of clothing or linen for the use of the infirmary. Grants of aid in renewing stocks of linen are made from the funds, which are in a flourishing condition. The president, Lady Susan Fortescue, sounded a note of warning in her address against allowing the girls, who were now doing good service in various directions, to fall into difficulties when the war is over for want of proper training. It is not too early to take steps to prevent this disaster, and Lady Susan was well advised in stepping outside her subject to lay stress on the need for looking ahead. Many matrons are giving attention already to the future prospects of the V.A.D., but too much indifference prevails generally on this subject.

☛ For the Registration of Nurses, by "Ierne," see p. 326; College of Nursing in Scotland London Hospital Nurses, p. 333; Matrons' and Sisters' Appointments, p. iv.

